# Supplementary figures and images for: Limited and idiosyncratic thermal acclimation in soil saprotrophic fungi
Source: PLoS One. 2026 May 27;21(5):e0349388. doi: 10.1371/journal.pone.0349388 (PMC13215554; doi:10.1371/journal.pone.0349388)

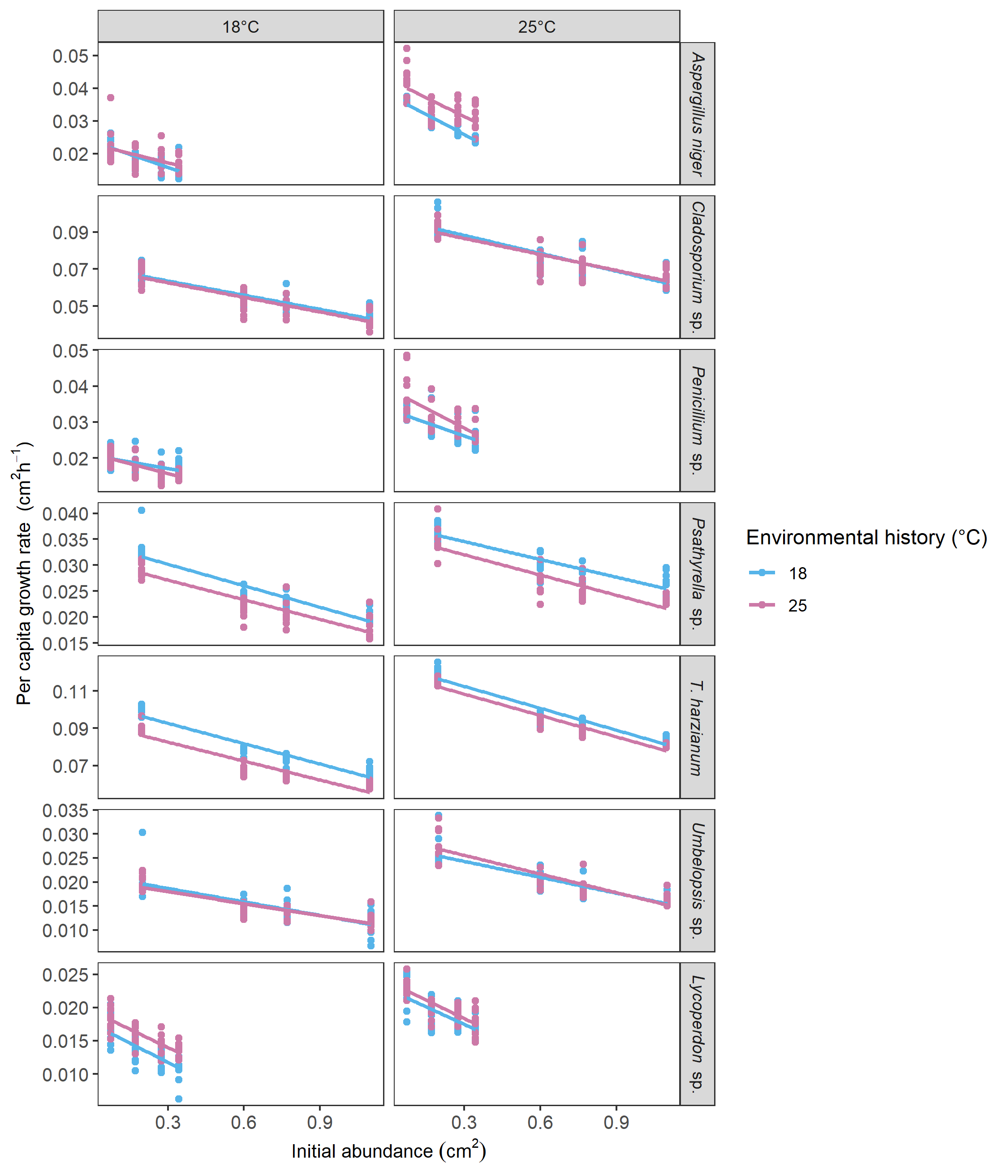

Supplement: S1 Fig — The columns represent each level of exposure temperature and the rows the different strains. (TIF) [file pone.0349388.s001.tif]

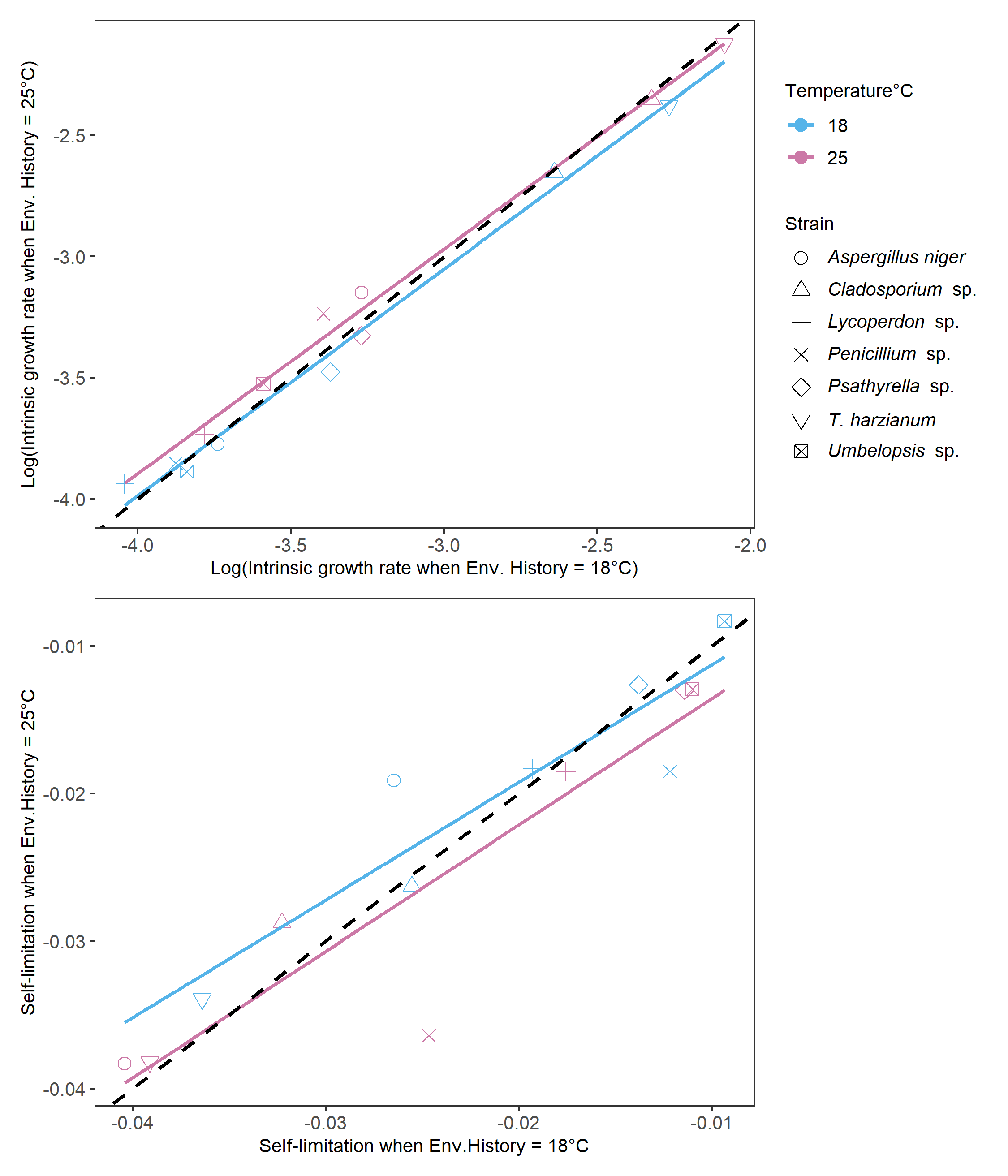

Supplement: S2 Fig — Both variables are log transformed. Bottom: Self-limitation when environmental history is 25ºC compared to the self-limitation when environmental history is 18ºC. Both plots: each strain is represented by two points: one at exposure temperature is 18ºC and another one when exposure temperature is 25ºC. (TIF) [file pone.0349388.s002.tif]

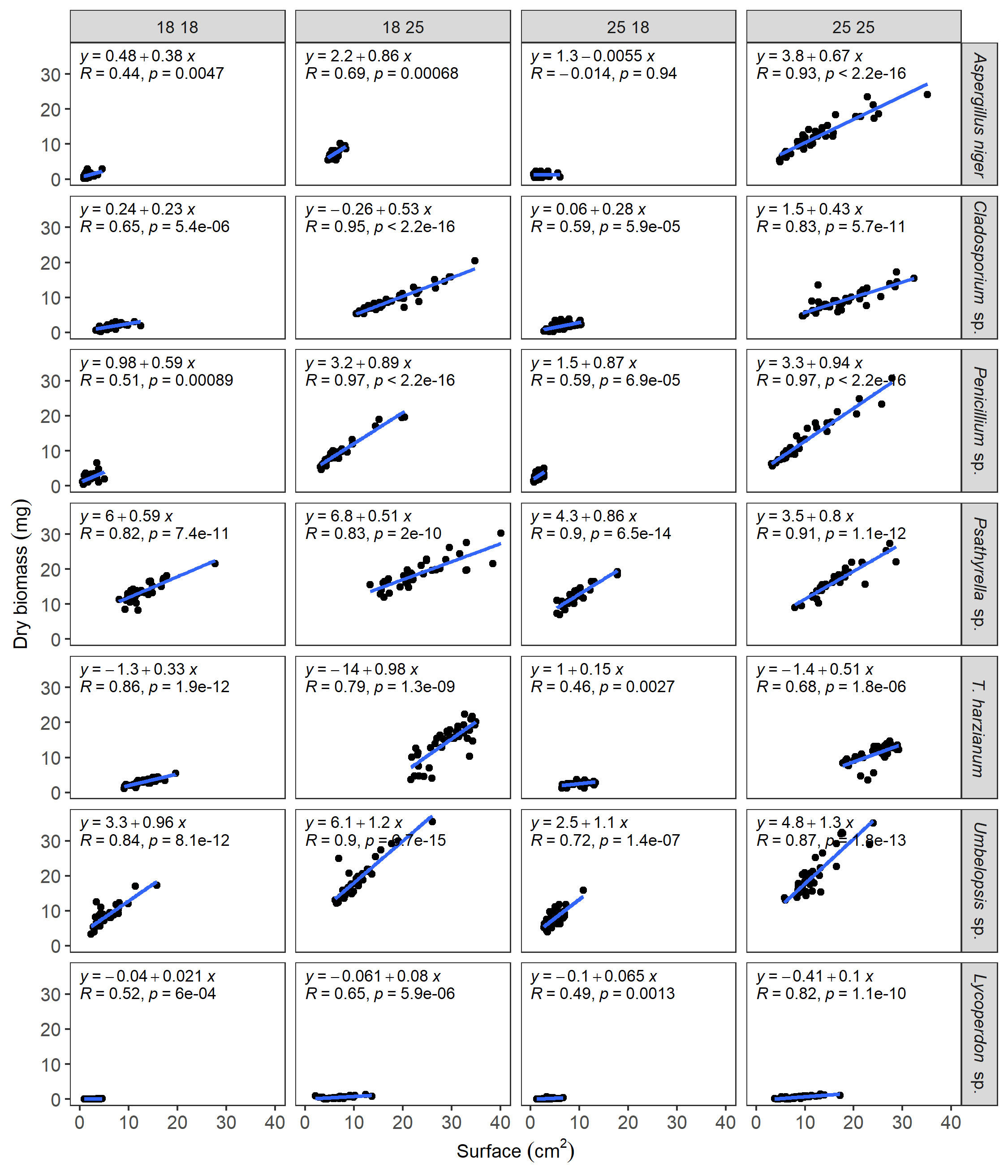

Supplement: S3 Fig — Each column is one treatment (including environmental history and exposure temperature) and each row is a strain. (TIF) [file pone.0349388.s003.tif]

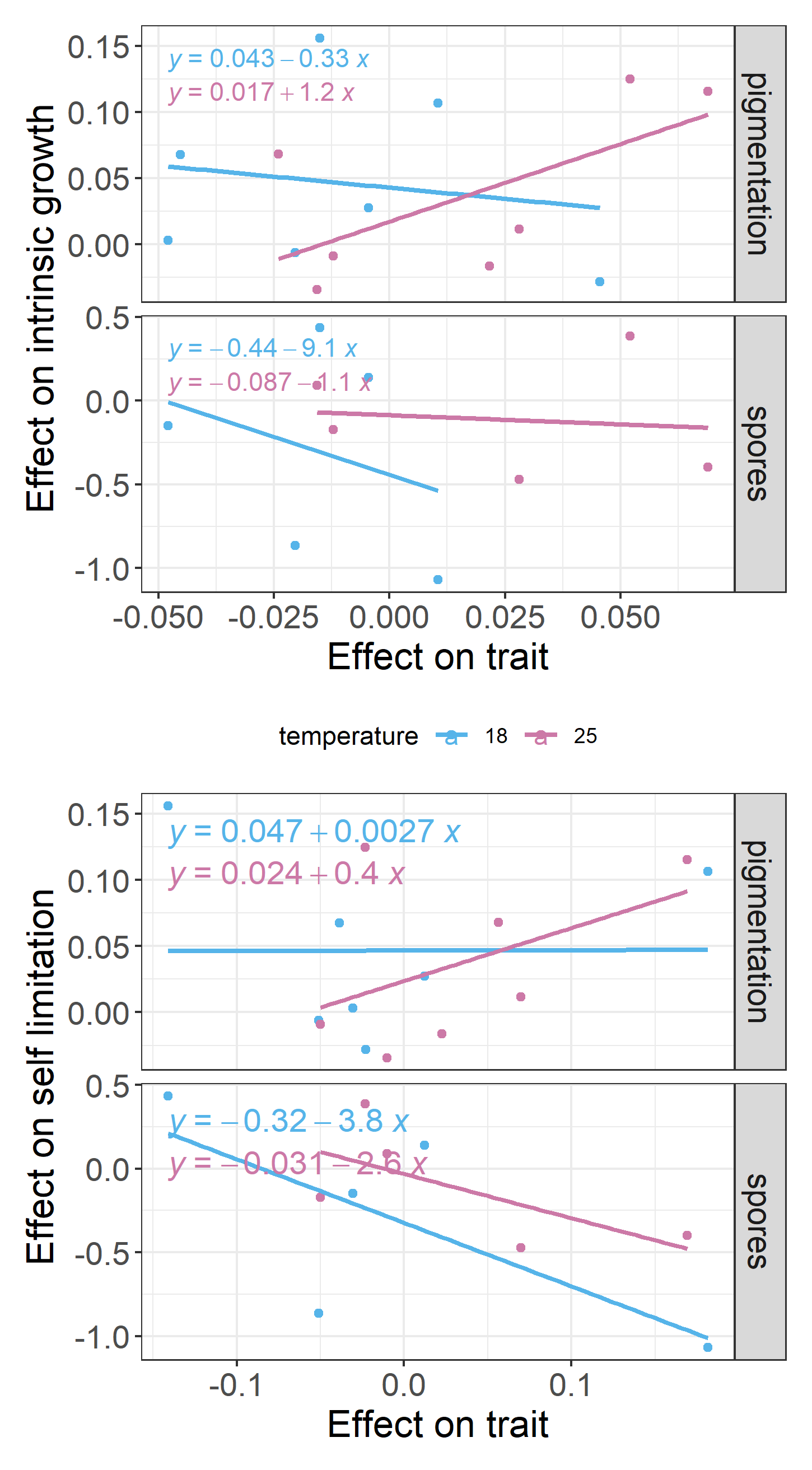

Supplement: S4 Fig — Each dot (seven dots per temperature in pigmentation panels and five points in the spore panels) represents one strain, so there are two points per strain in each plot (one per temperature). Spearman’s rank correlation results: growth ratio with pigmentation (18°C: ρ = 0, p = 1; 25°C: ρ = 0.43, p = 0.3536), growth ratio with spore load (18°C: ρ = -0.2, p = 0.7833; 25°C: ρ = -0.3, p = 0.6833), self-limitation with pigmentation (18°C: ρ = -0.18, p = 0.7131; 25°C: ρ = 0.036, p = 0.9635), and self-limitation with spore load (18°C: ρ = -0.6, p = 0.35; 25°C: ρ = -0.9, p = 0.0833). (TIF) [file pone.0349388.s004.tif]
