# Supplementary material for: Limited and idiosyncratic thermal acclimation in soil saprotrophic fungi
Source: PLoS One. 2026 May 27;21(5):e0349388. doi: 10.1371/journal.pone.0349388 (PMC13215554; doi:10.1371/journal.pone.0349388)
Supplement: S1 Table — We tested effects of environmental history, exposure temperature, and interactions on intrinsic growth and self limitation per strain. (DOCX) [file pone.0349388.s005.docx]

**Table S1.**

| **Strain** | **Factor** | **Df** | **Sum Sq** | **Mean Sq** | **F value** | **Pr(>F)** |
| --- | --- | --- | --- | --- | --- | --- |
| ***Trichoderma harzianum*** | Initial Density | 1 | 0.02 | 0.02 | 1258.37 | <0.001 |
|  | Environmental History | 1 | 0 | 0 | 92.36 | <0.001 |
|  | Exposure Temperature | 1 | 0.02 | 0.02 | 1015.62 | <0.001 |
|  | Initial Density:Environmental History | 1 | 0 | 0 | 0.67 | 0.41 |
|  | Initial Density:Exposure Temperature | 1 | 0 | 0 | 2.81 | 0.1 |
|  | Environmental History:Exposure Temperature | 1 | 0 | 0 | 15.8 | <0.001 |
|  | Initial Density:Environmental History:Exposure Temperature | 1 | 0 | 0 | 0.14 | 0.71 |
|  | Residuals | 152 | 0 | 0 |  |  |
| ***Cladosporium* sp.** | Initial Density | 1 | 0.01 | 0.01 | 375.4 | <0.001 |
|  | Environmental History | 1 | 0 | 0 | 0.71 | 0.4 |
|  | Exposure Temperature | 1 | 0.02 | 0.02 | 573.11 | <0.001 |
|  | Initial Density:Environmental History | 1 | 0 | 0 | 0.23 | 0.64 |
|  | Initial Density:Exposure Temperature | 1 | 0 | 0 | 2.51 | 0.12 |
|  | Environmental History:Exposure Temperature | 1 | 0 | 0 | 0.2 | 0.65 |
|  | Initial Density:Environmental History:Exposure Temperature | 1 | 0 | 0 | 0.53 | 0.47 |
|  | Residuals | 152 | 0.01 | 0 |  |  |
| ***Umbelopsis* sp.** | Initial Density | 1 | 0 | 0 | 352.84 | <0.001 |
|  | Environmental History | 1 | 0 | 0 | 0.14 | 0.71 |
|  | Exposure Temperature | 1 | 0 | 0 | 229.58 | <0.001 |
|  | Initial Density:Environmental History | 1 | 0 | 0 | 0.16 | 0.69 |
|  | Initial Density:Exposure Temperature | 1 | 0 | 0 | 7.97 | 0.01 |
|  | Environmental History:Exposure Temperature | 1 | 0 | 0 | 1.43 | 0.23 |
|  | Initial Density:Environmental History:Exposure Temperature | 1 | 0 | 0 | 1.78 | 0.18 |
|  | Residuals | 152 | 0 | 0 |  |  |
| ***Aspergillus niger*** | Initial Density | 1 | 0 | 0 | 93.67 | <0.001 |
|  | Environmental History | 1 | 0 | 0 | 67.37 | <0.001 |
|  | Exposure Temperature | 1 | 0.01 | 0.01 | 490.55 | <0.001 |
|  | Initial Density:Environmental History | 1 | 0 | 0 | 0.15 | 0.7 |
|  | Initial Density:Exposure Temperature | 1 | 0 | 0 | 7.87 | 0.01 |
|  | Environmental History:Exposure Temperature | 1 | 0 | 0 | 12.4 | <0.001 |
|  | Initial Density:Environmental History:Exposure Temperature | 1 | 0 | 0 | 0.17 | 0.68 |
| ***Penicillium* sp.** | Initial Density | 1 | 0 | 0 | 88.29 | <0.001 |
|  | Environmental History | 1 | 0 | 0 | 2.29 | 0.13 |
|  | Exposure Temperature | 1 | 0.01 | 0.01 | 529.24 | <0.001 |
|  | Initial Density:Environmental History | 1 | 0 | 0 | 2.91 | 0.09 |
|  | Initial Density:Exposure Temperature | 1 | 0 | 0 | 10.1 | 0.002 |
|  | Environmental History:Exposure Temperature | 1 | 0 | 0 | 14.76 | <0.001 |
|  | Initial Density:Environmental History:Exposure Temperature | 1 | 0 | 0 | 0.26 | 0.61 |
| ***Psathyrella* sp.** | Initial Density | 1 | 0 | 0 | 426.42 | <0.001 |
|  | Environmental History | 1 | 0 | 0 | 54.59 | <0.001 |
|  | Exposure Temperature | 1 | 0 | 0 | 159.66 | <0.001 |
|  | Initial Density:Environmental History | 1 | 0 | 0 | 0.01 | 0.93 |
|  | Initial Density:Exposure Temperature | 1 | 0 | 0 | 0.9 | 0.35 |
|  | Environmental History:Exposure Temperature | 1 | 0 | 0 | 0.31 | 0.58 |
|  | Initial Density:Environmental History:Exposure Temperature | 1 | 0 | 0 | 1.21 | 0.27 |
| ***Lycoperdon* sp.** | Initial Density | 1 | 0 | 0 | 183.91 | <0.001 |
|  | Environmental History | 1 | 0 | 0 | 32.34 | <0.001 |
|  | Exposure Temperature | 1 | 0.01 | 0.01 | 331.62 | <0.001 |
|  | Initial Density:Environmental History | 1 | 0 | 0 | 0 | 0.99 |
|  | Initial Density:Exposure Temperature | 1 | 0 | 0 | 0.1 | 0.75 |
|  | Environmental History:Exposure Temperature | 1 | 0 | 0 | 4.7 | 0.03 |
|  | Initial Density:Environmental History:Exposure Temperature | 1 | 0 | 0 | 0.13 | 0.72 |
