# Supplementary material for: Limited and idiosyncratic thermal acclimation in soil saprotrophic fungi
Source: PLoS One. 2026 May 27;21(5):e0349388. doi: 10.1371/journal.pone.0349388 (PMC13215554; doi:10.1371/journal.pone.0349388)
Supplement: S2 Table — We tested the effects of surface, environmental history, exposure temperature, and interactions on biomass. (DOCX) [file pone.0349388.s006.docx]

**Table S2**

| **Strain** | **Effect** | **Df** | **Sum Sq** | **Mean Sq** | **F value** | **Pr(>F)** |
| --- | --- | --- | --- | --- | --- | --- |
| ***Trichoderma harzianum*** | surface | 1 | 4653.3 | 4653.3 | 1302.49 | < 0.001 |
|  | Environmental history | 1 | 22.8 | 22.8 | 6.38 | 0.01 |
|  | Exposure temperature | 1 | 0.1 | 0.1 | 0.02 | 0.9 |
|  | surface:Environmental history | 1 | 91.1 | 91.1 | 25.5 | < 0.001 |
|  | surface:Exposure temperature | 1 | 51.4 | 51.4 | 14.4 | 0.0002 |
|  | Environmental history:Exposure temperature | 1 | 44.5 | 44.5 | 12.47 | 0.0005 |
|  | surface:Environmental history:Exposure temperature | 1 | 5.4 | 5.4 | 1.5 | 0.22 |
| ***Cladosporium* sp.** | surface | 1 | 2845.7 | 2845.7 | 2245.69 | < 0.001 |
|  | Environmental history | 1 | 0.35 | 0.35 | 0.27 | 0.6 |
|  | Exposure temperature | 1 | 72.82 | 72.82 | 57.47 | < 0.001 |
|  | surface:Environmental history | 1 | 4.48 | 4.48 | 3.54 | 0.06 |
|  | surface:Exposure temperature | 1 | 15.12 | 15.12 | 11.93 | 0.0007 |
|  | Environmental history:Exposure temperature | 1 | 1.98 | 1.98 | 1.57 | 0.21 |
|  | surface:Environmental history:Exposure temperature | 1 | 1.58 | 1.58 | 1.25 | 0.27 |
| ***Umbelopsis* sp.** | surface | 1 | 6943.5 | 6943.5 | 1493.96 | < 0.001 |
|  | Environmental history | 1 | 0.8 | 0.8 | 0.18 | 0.68 |
|  | Exposure temperature | 1 | 355.2 | 355.2 | 76.42 | < 0.001 |
|  | surface:Environmental history | 1 | 2.9 | 2.9 | 0.62 | 0.43 |
|  | surface:Exposure temperature | 1 | 16.7 | 16.7 | 3.59 | 0.06 |
|  | Environmental history:Exposure temperature | 1 | 2.2 | 2.2 | 0.48 | 0.49 |
|  | surface:Environmental history:Exposure temperature | 1 | 0 | 0 | 0.01 | 0.93 |
| ***Aspergillus niger*** | surface | 1 | 3824.1 | 3824.1 | 3128.89 | < 0.001 |
|  | Environmental history | 1 | 0.5 | 0.5 | 0.4 | 0.53 |
|  | Exposure temperature | 1 | 223.1 | 223.1 | 182.57 | < 0.001 |
|  | surface:Environmental history | 1 | 2.5 | 2.5 | 2.03 | 0.16 |
|  | surface:Exposure temperature | 1 | 23.4 | 23.4 | 19.12 | < 0.001 |
|  | Environmental history:Exposure temperature | 1 | 3.5 | 3.5 | 2.86 | 0.09 |
|  | surface:Environmental history:Exposure temperature | 1 | 0.3 | 0.3 | 0.28 | 0.6 |
| ***Penicillium* sp.** | surface | 1 | 4387.5 | 4387.5 | 3518.88 | < 0.001 |
|  | Environmental history | 1 | 13 | 13 | 10.44 | 0.002 |
|  | Exposure temperature | 1 | 131.5 | 131.5 | 105.43 | < 0.001 |
|  | surface:Environmental history | 1 | 0 | 0 | 0.03 | 0.86 |
|  | surface:Exposure temperature | 1 | 3.9 | 3.9 | 3.14 | 0.08 |
|  | Environmental history:Exposure temperature | 1 | 3.5 | 3.5 | 2.78 | 0.1 |
|  | surface:Environmental history:Exposure temperature | 1 | 0.6 | 0.6 | 0.46 | 0.5 |
| ***Psathyrella* sp.** | surface | 1 | 2168.8 | 2168.8 | 729.56 | < 0.001 |
|  | Environmental history | 1 | 49.7 | 49.7 | 16.71 | < 0.001 |
|  | Exposure temperature | 1 | 8.74 | 8.74 | 2.94 | 0.09 |
|  | surface:Environmental history | 1 | 66.7 | 66.7 | 22.43 | < 0.001 |
|  | surface:Exposure temperature | 1 | 0.87 | 0.87 | 0.29 | 0.59 |
|  | Environmental history:Exposure temperature | 1 | 7.18 | 7.18 | 2.42 | 0.12 |
|  | surface:Environmental history:Exposure temperature | 1 | 0.06 | 0.06 | 0.02 | 0.89 |
| ***Lycoperdon* sp.** | surface | 1 | 11.4 | 11.4 | 360.37 | < 0.001 |
|  | Environmental history | 1 | 0.16 | 0.16 | 5.13 | 0.03 |
|  | Exposure temperature | 1 | 0 | 0 | 0 | 0.99 |
|  | surface:Environmental history | 1 | 0.03 | 0.03 | 0.88 | 0.35 |
|  | surface:Exposure temperature | 1 | 0.05 | 0.05 | 1.72 | 0.19 |
|  | Environmental history:Exposure temperature | 1 | 0.52 | 0.52 | 16.43 | < 0.001 |
|  | surface:Environmental history:Exposure temperature | 1 | 0.01 | 0.01 | 0.21 | 0.65 |
