# Supplementary material for: Limited and idiosyncratic thermal acclimation in soil saprotrophic fungi
Source: PLoS One. 2026 May 27;21(5):e0349388. doi: 10.1371/journal.pone.0349388 (PMC13215554; doi:10.1371/journal.pone.0349388)
Supplement: S1 Text — Detailed description of protocols used to culture and maintain fungal strains for the experiment. (DOCX) [file pone.0349388.s007.docx]

**S1 Text.**

**Protocol description**

Strains were stored at 5°C between experiments. Prior to starting the experiment, we reactivated them on the experimental medium at 25°C for at least one week, which is a standard protocol in fungal experimental work [1,2]. This reactivation step ensures that strains are metabolically active and viable before exposure to the experimental treatments.

**Possible consequences and clarification**

We acknowledge that reactivation at 25°C could, in theory, influence the initial physiology of the strains, particularly for those later transferred to 18°C, where a short lag phase may occur. However, this does not compromise our results for two reasons: (i) reactivation at room temperature (22-25°C) is a common and widely accepted procedure in fungal studies, ensuring comparability with previous work; and (ii) any initial lag phase would be minor and transient relative to the duration of the experiment, meaning that the observed responses reflect the experimental treatments rather than artifacts of reactivation.

**References**

1. Hare J. Sabouraud Agar for Fungal Growth Protocols. American Society for Microbiology; 2008. Available: https://asm.org/protocols/sabouraud-agar-for-fungal-growth-protocols

2. Richter DL. Revival of saprotrophic and mycorrhizal basidiomycete cultures after 20 years in cold storage in sterile water. Can J Microbiol. 2008;54: 595–599. doi:10.1139/W08-049
