# Supplementary material for: Limited and idiosyncratic thermal acclimation in soil saprotrophic fungi
Source: PLoS One. 2026 May 27;21(5):e0349388. doi: 10.1371/journal.pone.0349388 (PMC13215554; doi:10.1371/journal.pone.0349388)
Supplement: Attachment — Submitted filename: Response to Reviewers.docx [file pone.0349388.s008.docx]

1. When submitting your revision, we need you to address these additional requirements.

Please ensure that your manuscript meets PLOS ONE's style requirements, including those for file naming. The PLOS ONE style templates can be found at

<https://journals.plos.org/plosone/s/file?id=wjVg/PLOSOne_formatting_sample_main_body.pdf> and

<https://journals.plos.org/plosone/s/file?id=ba62/PLOSOne_formatting_sample_title_authors_affiliations.pdf>

**The manuscript files have been updated.**

2. Thank you for stating in your Funding Statement:

This study was financially supported by BOF (Bijzonder Onderzoeksfonds) and FSR (Fonds Spécial de Recherche) (both Special Research Funds) from Hasselt University and Namur University, respectively, under the research grant number BOF21DOCNA01

BOF-FSR URL: <https://www.uhasselt.be/en/calls-research/detail/1195-2023-bof-joint-phd-programme-unamur-and-uhasselt>

The funders had no role in study design, data collection and analysis, decision to publish, or preparation of the manuscript

Please provide an amended statement that declares *all* the funding or sources of support (whether external or internal to your organization) received during this study, as detailed online in our guide for authors at <http://journals.plos.org/plosone/s/submit-now>.  Please also include the statement “There was no additional external funding received for this study.” in your updated Funding Statement.

Please include your amended Funding Statement within your cover letter. We will change the online submission form on your behalf.

**We have amended the Funding Statement and it has been included in the cover letter, as requested.**

3. In the online submission form, you indicated that your data will be submitted to a repository upon acceptance.  We strongly recommend all authors deposit their data before acceptance, as the process can be lengthy and hold up publication timelines. Please note that, though access restrictions are acceptable now, your entire minimal  dataset will need to be made freely accessible if your manuscript is accepted for publication. This policy applies to all data except where public deposition would breach compliance with the protocol approved by your research ethics board. If you are unable to adhere to our open data policy, please kindly revise your statement to explain your reasoning and we will seek the editor's input on an exemption.

**The dataset and associated R scripts supporting this study have been deposited in Zenodo and are currently available to the reviewers via a private access link:** [**private access to reviewers**](https://zenodo.org/records/15010903?preview=1&token=eyJhbGciOiJIUzUxMiJ9.eyJpZCI6IjU4ZGQ4ODQ1LWZlNGEtNDdiMS05YjExLWFlZjM3NTE3NjFmMyIsImRhdGEiOnt9LCJyYW5kb20iOiI3YzQyYmEzYTBjZjYzMmQ0MzBhOTVmNWFkMTJlMDUxYSJ9.OTUJ-r2cH_yU9fojuU2n064lWJwJfuuhqF1FEtyRNVv4MTvdH3XYcyyNNuMDF4Z7fh7uNQiS6fYOsserwHJwOw). **A permanent Zenodo DOI has been reserved and will be activated upon acceptance of the manuscript, at which point the complete dataset and all analysis code will be made freely and publicly available without restriction, in full accordance with PLOS ONE’s open data policy.**

**Reserved DOI: https://doi.org/10.5281/zenodo.15010903**

4. When completing the data availability statement of the submission form, you indicated that you will make your data available on acceptance. We strongly recommend all authors decide on a data sharing plan before acceptance, as the process can be lengthy and hold up publication timelines. Please note that, though access restrictions are acceptable now, your entire data will need to be made freely accessible if your manuscript is accepted for publication. This policy applies to all data except where public deposition would breach compliance with the protocol approved by your research ethics board. If you are unable to adhere to our open data policy, please kindly revise your statement to explain your reasoning and we will seek the editor's input on an exemption. Please be assured that, once you have provided your new statement, the assessment of your exemption will not hold up the peer review process.

**Same answer as in previous question.**

5. Please upload a new copy of Figure 2, S1, S2, S3 and S4, as the detail is not clear. Please follow the link for more information:  <https://journals.plos.org/plosone/s/figures>

**We have uploaded new, high-resolution versions of Figure 2 and Supplementary Figures S1-S4, following PLOS ONE’s guidelines.**

6. If the reviewer comments include a recommendation to cite specific previously published works, please review and evaluate these publications to determine whether they are relevant and should be cited. There is no requirement to cite these works unless the editor has indicated otherwise. 

7. Please review your reference list to ensure that it is complete and correct. If you have cited papers that have been retracted, please include the rationale for doing so in the manuscript text, or remove these references and replace them with relevant current references. Any changes to the reference list should be mentioned in the rebuttal letter that accompanies your revised manuscript. If you need to cite a retracted article, indicate the article’s retracted status in the References list and also include a citation and full reference for the retraction notice.

**The reference list has been carefully reviewed and corrected where necessary. In-text citations have been updated where necessary to follow PLOS ONE’s numbering format.**

Reviewer's Responses to Questions

Comments to the Author

1. Is the manuscript technically sound, and do the data support the conclusions?

The manuscript must describe a technically sound piece of scientific research with data that supports the conclusions. Experiments must have been conducted rigorously, with appropriate controls, replication, and sample sizes. The conclusions must be drawn appropriately based on the data presented.

Reviewer #1: Yes

2. Has the statistical analysis been performed appropriately and rigorously?

Reviewer #1: Yes

3. Have the authors made all data underlying the findings in their manuscript fully available?

The [PLOS Data policy](http://track.editorialmanager.com/CL0/http:%2F%2Fwww.plosone.org%2Fstatic%2Fpolicies.action%23sharing/1/010f019be4554dbf-8c103a30-f06d-4b49-a417-f27644151bb7-000000/7aUs8S-5B_FKIC6P2Moh-FPpxAYA6ibFPfLYBSPJKdw=247) requires authors to make all data underlying the findings described in their manuscript fully available without restriction, with rare exception (please refer to the Data Availability Statement in the manuscript PDF file). The data should be provided as part of the manuscript or its supporting information, or deposited to a public repository. For example, in addition to summary statistics, the data points behind means, medians and variance measures should be available. If there are restrictions on publicly sharing data—e.g. participant privacy or use of data from a third party—those must be specified.

Reviewer #1: Yes

4. Is the manuscript presented in an intelligible fashion and written in standard English?

PLOS ONE does not copyedit accepted manuscripts, so the language in submitted articles must be clear, correct, and unambiguous. Any typographical or grammatical errors should be corrected at revision, so please note any specific errors here.

Reviewer #1: Yes

5. Review Comments to the Author

Please use the space provided to explain your answers to the questions above. You may also include additional comments for the author, including concerns about dual publication, research ethics, or publication ethics. (Please upload your review as an attachment if it exceeds 20,000 characters)

Reviewer #1: The study presents original experimental work involving seven saprobic fungal species and examines the combined effects of thermal history and thermal exposure across two experimental phases. The integration of intrinsic growth, self-limitation, pigmentation, and spore load as response variables is innovative and adds depth to the understanding of microbial thermal acclimation. This constitutes a meaningful contribution to the field.

**We sincerely thank the reviewer for the positive and constructive evaluation of our work.**

2. Prior Publication
No issues were identified regarding prior publication or duplication of results. The dataset and analyses appear to be novel and specific to this study.

3. Experimental Design and Statistical Analyses
The experimental design is generally strong, with adequate replication (10 replicates per treatment), ecologically relevant temperatures, and appropriate use of linear regression to estimate growth parameters.
However, there are a few issues that require correction for clarity:
• In the description accompanying Figure 1 (lines 140 and 142), there is a formatting error in one of the initial density values (“0. 17” contains an extraneous space).
• The list of species associated with initial densities presents inconsistent punctuation (e.g., “Cladosporium sp., Psathyrella sp.; Trichoderma harzianum and Umbelopsis sp.”).
This may cause confusion regarding which species correspond to which density values.
Suggestion:
Please revise this section to correct numerical formatting and standardize punctuation and grouping of species.
**We have corrected the numerical formatting error and the punctuation inconsistencies.**

4. Conclusions
The conclusions are well supported by the data. The findings that thermal acclimation was limited and species-dependent, that pigmentation and spore load did not predict acclimation, and that effects were generally modest (<20%) are all consistent with the analyses presented. The interpretation is balanced and does not overstate the implications.

5. Manuscript Clarity and Language
The manuscript is clearly written and the English is generally very good. Some minor improvements related to punctuation and sentence flow could help improve readability, but these are optional and do not impede understanding.

**We have carefully reviewed the manuscript and implemented minor edits to improve punctuation and sentence flow where appropriate, without altering the scientific content.**

6. Ethics and Research Transparency
Although no ethical concerns are associated with the use of saprobic fungi, I recommend including explicit information on:
• The origin of the natural samples used.
• Whether any collection permits were required and obtained.
This would improve transparency, particularly for readers evaluating reproducibility.

**We have added information on the origin of the natural samples in Table 1 and clarified whether collection permits were obtained in the “Culturing fungal strains” subsection of the Materials and Methods**

7. Reporting Standards and Data Availability
The manuscript appears to follow appropriate reporting guidelines, and the data availability statement meets journal requirements.

Overall Assessment
This is a solid and well-executed study with clear relevance to microbial ecology and thermal biology. Only minor revisions are needed, primarily related to formatting and clarification in specific parts of the Methods section. Once these points are addressed, the manuscript will be suitable for publication.

6. PLOS authors have the option to publish the peer review history of their article ([what does this mean?](https://track.editorialmanager.com/CL0/https:%2F%2Fjournals.plos.org%2Fplosone%2Fs%2Feditorial-and-peer-review-process%23loc-peer-review-history/1/010f019be4554dbf-8c103a30-f06d-4b49-a417-f27644151bb7-000000/OdySWlmMHDTkJRx-t9phktm-lH5ZzlOKWVeKkiCOHy4=247)). If published, this will include your full peer review and any attached files.


If you choose “no”, your identity will remain anonymous but your review may still be made public.


Do you want your identity to be public for this peer review? For information about this choice, including consent withdrawal, please see our [Privacy Policy](https://track.editorialmanager.com/CL0/https:%2F%2Fwww.plos.org%2Fprivacy-policy/1/010f019be4554dbf-8c103a30-f06d-4b49-a417-f27644151bb7-000000/8_gbQ5skKQ9oLXhdHk13jOPio3qw8sFwJ1pvHQZdKPQ=247).

Reviewer #1: No
