# Supplementary material for: Limited and idiosyncratic thermal acclimation in soil saprotrophic fungi
Source: PLoS One. 2026 May 27;21(5):e0349388. doi: 10.1371/journal.pone.0349388 (PMC13215554; doi:10.1371/journal.pone.0349388)
Supplement: Attachment — Submitted filename: Response_to_Reviewers_auresp_2.docx [file pone.0349388.s009.docx]

**Response to Reviewers**

**Note on Line Numbers: Please note that all line numbers mentioned in our responses below refer to the clean version of the revised manuscript (the file labeled 'Manuscript').**

Reviewers' comments:

Reviewer's Responses to Questions

Comments to the Author

1. If the authors have adequately addressed your comments raised in a previous round of review and you feel that this manuscript is now acceptable for publication, you may indicate that here to bypass the “Comments to the Author” section, enter your conflict of interest statement in the “Confidential to Editor” section, and submit your "Accept" recommendation.

Reviewer #2: All comments have been addressed

Reviewer #3: (No Response)

2. Is the manuscript technically sound, and do the data support the conclusions?

The manuscript must describe a technically sound piece of scientific research with data that supports the conclusions. Experiments must have been conducted rigorously, with appropriate controls, replication, and sample sizes. The conclusions must be drawn appropriately based on the data presented.

Reviewer #2: Yes

Reviewer #3: Partly

3. Has the statistical analysis been performed appropriately and rigorously?

Reviewer #2: Yes

Reviewer #3: Yes

4. Have the authors made all data underlying the findings in their manuscript fully available?

The [PLOS Data policy](http://track.editorialmanager.com/CL0/http:%2F%2Fwww.plosone.org%2Fstatic%2Fpolicies.action%23sharing/1/010f019d27ca3598-9f9ad00c-08ca-4854-9c45-09ebbf9a979a-000000/_yLEWvdpD6_GULt_tOjI0WGjyOo3YXnUpbPZwrOzLQk=256) requires authors to make all data underlying the findings described in their manuscript fully available without restriction, with rare exception (please refer to the Data Availability Statement in the manuscript PDF file). The data should be provided as part of the manuscript or its supporting information, or deposited to a public repository. For example, in addition to summary statistics, the data points behind means, medians and variance measures should be available. If there are restrictions on publicly sharing data—e.g. participant privacy or use of data from a third party—those must be specified.

Reviewer #2: Yes

Reviewer #3: Yes

5. Is the manuscript presented in an intelligible fashion and written in standard English?

PLOS ONE does not copyedit accepted manuscripts, so the language in submitted articles must be clear, correct, and unambiguous. Any typographical or grammatical errors should be corrected at revision, so please note any specific errors here.

Reviewer #2: Yes

Reviewer #3: Yes

6. Review Comments to the Author

Please use the space provided to explain your answers to the questions above. You may also include additional comments for the author, including concerns about dual publication, research ethics, or publication ethics. (Please upload your review as an attachment if it exceeds 20,000 characters)

**We thank Reviewer #2 for their helpful comments. We have implemented the requested changes as detailed below.**

Reviewer #2: Figure 2: Please make the left and right panel A and B, respectively.

**We have corrected this in the figure and in the caption of the figure (Line 294).**

Methods line 149-150: Please explicitly state why initial density ranges differed by strain.

**We used different initial densities to create a density range in which growth is expected to respond to density, which we needed to measure density-dependent growth. A common density range across all strains would not have been appropriate, because it could be too high for slower-growing strains (yielding consistently negative growth) or too low for faster-growing strains (yielding consistently positive growth), reducing our ability to estimate the density-dependence regression. We clarify this on Lines 153-157.**

Reviewer #3: This manuscript examines whether common soil saprotrophic fungi can acclimate to realistic temperature increases associated with heatwaves, and whether key traits, such as pigmentation and spore load, can predict this capacity. In general, the authors use a well-designed experiment to address a knowledge gap in microbial feedback to climate change. However, there are issues in experimental design, data interpretation, and presentation that need to be addressed to support the conclusions.

**We thank Reviewer #3 for their supportive and helpful comments. We have carefully addressed the concerns in the revised manuscript. Our point-by-point responses to the specific suggestions follow below.**

The authors use “acclimation” as their central concept, but they fail to distinguish it from other forms of short-term phenotypic plasticity clearly. They indicate that they focus on “short-term responses (ranging from hours to days)” and therefore use the term “acclimation.” However, the experiment involves a one-week “environmental history” treatment, which does not correspond to a short-term period but rather to sustained exposure that could induce a range of responses. The authors need to define what constitutes “acclimation” in this context clearly.

**We sincerely appreciate this conceptual critique. We agree with the reviewer that ‘acclimation’ is a part of the broader concept of phenotypic plasticity. Thus, in the revised Introduction (Lines 49-55), we have clarified our definition. We use 'acclimation' to describe the stabilized phenotypic response that occurs when an organism is exposed to a new environment for a duration sufficient to reach a new physiological steady-state, but insufficient for evolutionary genetic change.**

The authors assess growth rates at 40-186 h to compare fast- and slow-growing strains. However, this could be misleading. For a given fast-growing strain (40 h), the measurement might capture exponential growth, whereas for a slow-growing strain (186 h), it might capture the transition to the stationary phase. The differences in the growth phases sampled could significantly affect estimates of the intrinsic growth rate and self-limitation, complicating comparisons between strains. The authors should clearly justify that the selected time points are all within the same phase of population growth for each strain. If this cannot be established, they should acknowledge it as a significant limitation of the study.

**We thank the reviewer for this important observation. However, the goal is not to compare absolute growth among strains. Instead, the goal is to regress the instantaneous per-capita growth rate against the corresponding initial density to estimate density-dependent growth. Instead of measuring directly the instantaneous per-capita growth rate (**$\frac{\mathbf{d}\boldsymbol{N}}{\mathbf{Nd}\boldsymbol{t}}$**) at some initial density** $\mathbf{N}$**, one must approximate it by measuring** $\boldsymbol{\Delta(}\ln\left( \mathbf{N} \right)\boldsymbol{)/\Delta t}$**, where**$\boldsymbol{\Delta(ln(N)}\boldsymbol{)}$ **is the increment of log-tranformed density over a time interval** $\boldsymbol{\Delta}\boldsymbol{t}$ **(e.g. 40h, 186h). This approximation gets more precise when the interval** $\boldsymbol{\Delta}\boldsymbol{t}$ **is shorter. However, if the interval is too short, density changes become too small to measure empirically, and the approximation of instantaneous growth would be swamped by measurement error. We therefore selected a strain-specific interval that we expected to be short enough to approximate instantaneous growth, but long enough to produce measurable biomass change. Because strains differed substantially in growth rate, slower-growing strains logically required longer intervals than faster-growing strains to achieve this. This is why the durations differed by strain. This wasn’t very clear, and we explain this now on Lines 182-189 & Lines 206-209.**

**Regarding density dependence: the purpose of varying initial density and approximating instantaneous growth was precisely to estimate density dependence. We acknowledge that we cannot exclude that Δt was too long, leading to a poorer approximation of the instantaneous per-capita growth rate. However, there is no reason to believe that this is more of an issue for the fast or slow strains, exactly because we accounted for this by varying the duration. This is acknowledged on Lines 192-194.**

The ANOVA in Table 2 is a key strength, and its interpretation is essential. In this analysis, strain alone accounts for 87% of the variance. The next largest effect is exposure temperature (6%), and the interaction of interest (Environmental History × Exposure Temperature) accounts for <0.1% of the variance. At this point, the authors highlight that the strain-specific responses to environmental history reported in the results section (lines 277–285) show some statistically significant effects; however, the magnitude of these effects is small relative to the overall model. Accordingly, the discussion should be rephrased to emphasize that, within the overall variance framework, environmental history is a minor factor compared to species identity and current temperature.

**We fully agree with the reviewer that the hierarchical importance of these factors must be clearly contextualized. While we found statistically significant interactions, the effect size clearly shows that strain identity (87%) and exposure temperature (6%) are the primary drivers of growth, with environmental history accounting for <0.1%.**

**We have revised the Results (Lines 313-318), ensuring the reader understands the relative scale of these effects. Furthermore, we have updated the Discussion (Lines 407-410 & Lines 418-420) and Conclusions (Lines 492-493) to emphasize that thermal acclimation serves as a 'minor modulation' rather than a primary driver of fungal population dynamics in this context. This shift in emphasis ensures that our biological interpretations align with the magnitude of the statistical effects.**

Lines 200-227: The authors measure pigmentation as "darkness" based on an area of 0.04 cm² at the edge of the mycelium. This is an approximate method. Pigment content can be quantified biochemically. The authors should justify this method and acknowledge its limitations. Similarly, spore load is a static measurement taken after treatment. Neither spore germination nor the proportion of biomass allocated to spores versus mycelium during the growth phase is considered.

**We have addressed these comments and acknowledged the limitations as follows:**

**Pigmentation: In the Material and Methods (Lines 233-236), we have justified the use of ‘darkness’ as a non-destructive proxy for pigmentation. We have also acknowledged that other biochemical methods are more accurate. In the Discussion (Lines 442-444), we acknowledge that while this method is effective for broad comparisons, its coarse nature compared to biochemical pigment extraction might have obscured subtle correlations.**

**Spore load: We have clarified in the Material and Methods (Lines 251-255) that our spore count represents a standardized measurement (spores*cm^-2^), taken as a ‘static snapshot’ at a specific time point.**

**To address the reviewer's point about biomass allocation to spores versus mycelium, we have expanded the Discussion (Lines 444-451). We explained that our spore measurement was analyzed independently of the total colony biomass. Due to the different scales and the non-uniform distribution of spores across the mycelium, a direct ratio of reproductive investment could not be reliably extrapolated for the entire individual. Therefore, we agree that our measurement prevents a full assessment of biomass allocation trade-offs, which may explain why our hypothesis was not supported.**

The authors claim that “the phenomenon of fungal acclimatization appears to have little, if any, impact on the decomposition processes of soil organic matter” (lines 382-386) is an excessive extrapolation. The study measured growth in a synthetic medium (MMN) under pure culture conditions. This is a far cry from the complex, competitive, and resource-limited environment of the soil. They measured growth, not the decomposition of complex organic matter such as lignin or cellulose. The authors must narrow this statement to center their conclusion on the simplified growth conditions evaluated in this in vitro study and acknowledge the need for further research under more ecologically relevant conditions.

**We agree that our initial phrasing was too broad and failed to sufficiently emphasize the simplified nature of our experimental setup (pure cultures on synthetic media).**

**We have revised the Discussion (Lines 423, 426- 429) to explicitly narrow our conclusions to the in vitro conditions evaluated. We now clearly state that while our results suggest a limited role for thermal acclimation in these strains, the breakdown of complex organic matter (like lignin) and the influence of interspecific competition in natural soil may lead to different outcomes. We have ensured that our Conclusions reflect the need for further field-scale research to bridge this gap (Lines 495-498)**

7. PLOS authors have the option to publish the peer review history of their article ([what does this mean?](https://track.editorialmanager.com/CL0/https:%2F%2Fjournals.plos.org%2Fplosone%2Fs%2Feditorial-and-peer-review-process%23loc-peer-review-history/1/010f019d27ca3598-9f9ad00c-08ca-4854-9c45-09ebbf9a979a-000000/38_Rx_yg_8sSChr9OBQDJ8n7z0C5pCDCKzflPq0art4=256)). If published, this will include your full peer review and any attached files.


If you choose “no”, your identity will remain anonymous but your review may still be made public.


**Do you want your identity to be public for this peer review?** For information about this choice, including consent withdrawal, please see our [Privacy Policy](https://track.editorialmanager.com/CL0/https:%2F%2Fwww.plos.org%2Fprivacy-policy/1/010f019d27ca3598-9f9ad00c-08ca-4854-9c45-09ebbf9a979a-000000/uGUw7FNp-lpzP4AyCCMi9MF45NEJM-kiyjspZkiV2Po=256).

Reviewer #2: No

Reviewer #3: No
